# The Long Non-Coding RNA *Prader Willi/Angelman Region RNA5* (*PAR5*) Is Downregulated in Anaplastic Thyroid Carcinomas Where It Acts as a Tumor Suppressor by Reducing EZH2 Activity

**DOI:** 10.3390/cancers12010235

**Published:** 2020-01-17

**Authors:** Simona Pellecchia, Romina Sepe, Myriam Decaussin-Petrucci, Cristina Ivan, Masayoshi Shimizu, Carmela Coppola, Domenico Testa, George Adrian Calin, Alfredo Fusco, Pierlorenzo Pallante

**Affiliations:** 1 Institute for Experimental Endocrinology and Oncology (IEOS) “G. Salvatore”, National Research Council (CNR), via S. Pansini, 5-80131 Naples, Italy; simona.pellecchia@gmail.com (S.P.); romina.sepe@unina.it (R.S.); 2 Department of Molecular Medicine and Medical Biotechnology (DMMBM), University of Naples “Federico II” via S. Pansini, 5-80131 Naples, Italy; 3 Service d’Anatomie et Cytologie Pathologiques, Centre de Biologie Sud, Groupement Hospitalier Lyon Sud, Universite Lyon 1, 69495 Pierre Bénite, France; myriam.decaussin-petrucci@chu-lyon.fr; 4 Department of Experimental Therapeutics, The University of Texas MD Anderson Cancer Center, Houston, TX 77030, USA; CIvan@mdanderson.org (C.I.); mshimizu@mdanderson.org (M.S.); gcalin@mdanderson.org (G.A.C.); 5 Center for RNA Interference and Non-Coding RNAs, The University of Texas MD Anderson Cancer Center, Houston, TX 77030, USA; 6 Scientific Directorate, Istituto Nazionale Tumori di Napoli, IRCCS “G. Pascale”, via M. Semmola, 80131 Naples, Italy; c.coppola@istitutotumori.na.it; 7 Otorhinolaryngology, Head and Neck Surgery Unit, Department of Mental and Physical Health and Preventive Medicine, University of Campania “Luigi Vanvitelli”, via S. Pansini, 5-80131 Naples, Italy; domenico.testa@unicampania.it

**Keywords:** anaplastic thyroid carcinoma, EZH2, *PAR5*, long non-coding RNA

## Abstract

Anaplastic thyroid carcinoma (ATC) represents one the most aggressive neoplasias in humans, and, nowadays, limited advances have been made to extend the survival and reduce the mortality of ATC. Thus, the identification of molecular mechanism underlying its progression is needed. Here, we evaluated the long non-coding RNA (lncRNA) expression profile of nine ATC in comparison with five normal thyroid tissues by a lncRNA microarray. By this analysis, we identified 19 upregulated and 28 downregulated lncRNAs with a fold change >1.1 or <−1.1 and *p*-value < 0.05, in ATC samples. Some of them were subsequently validated by qRT-PCR. Then, we investigated the role of the lncRNA *Prader Willi/Angelman region RNA5* (*PAR5*), drastically and specifically downregulated in ATC. The restoration of *PAR5* reduces proliferation and migration rates of ATC-derived cell lines indicating that its downregulation contributes to thyroid cancer progression. Our results suggest that *PAR5* exerts its anti-oncogenic role by impairing Enhancer of Zeste Homolog 2 (EZH2) oncogenic activity since we demonstrated that *PAR5* interacts with it in thyroid cancer cell lines, reducing EZH2 protein levels and its binding on the *E-cadherin* promoter, relieving E-cadherin from the negative regulation by EZH2. Consistently, EZH2 is overexpressed in ATC, but not in differentiated thyroid carcinomas. The results reported here define a tumor suppressor role for *PAR5* in undifferentiated thyroid neoplasias, further highlighting the pivotal role of lncRNAs in thyroid carcinogenesis.

## 1. Introduction

Thyroid cancer (TC) is the most common endocrine malignancy, accounting for 1% of all human cancers, and its incidence has been significantly increased worldwide in the last decades [1]. Based on the histological features, thyroid carcinomas have been classified into well-differentiated papillary (PTC) and follicular thyroid carcinomas (FTC), poorly differentiated (PDC), and undifferentiated anaplastic thyroid carcinomas (ATC) [2]. Among them, ATC is less common but represents the most aggressive and lethal thyroid neoplasia with an overall survival rate of 3–5 months after diagnosis, being resistant to chemo- and radio-therapy [3].

It has been previously reported that p53 impairment represents a feature of ATC [4,5]. More recently, B-RAF mutations have been detected in about 30% of ATC [6,7]. Additionally, it is proven that ATC aggressiveness is further due to mutations of human telomerase reverse transcriptase (TERT) [8]. As far as epigenetic events in ATC are concerned, the Enhancer of Zeste Homolog 2 (EZH2), a member of the polycomb group (PcG) proteins, has been found overexpressed in ATC, but not PTC [9]. Moreover, a significant downregulation of miR-30d, miR-125b, miR-26a, and miR-30a-5p was detected in ATC samples in comparison to normal thyroid (NT) tissues [10].

However, the molecular bases of ATC development are still far from being understood, and, therefore, the unveiling of novel molecular mechanisms involved in ATC carcinogenesis is required to propose an efficient treatment for ATC. 

Long non-coding RNAs (lncRNAs) are a class of heterogeneous non-coding transcripts longer than 200 nucleotides [11]. Recently, it has been shown that lncRNAs play a pivotal role in several cellular processes such as differentiation, development, and cellular senescence [12,13]. Accumulating evidence implies that lncRNAs exert regulatory functions at both transcriptional and post-transcriptional levels [14,15,16]. Indeed, several studies have reported that lncRNAs interact with DNA, RNA, and protein to regulate protein function, induce chromatin remodeling and histone modification, acting as a scaffold, and modulate protein stability and DNA methylation [17,18,19]. The deregulation of lncRNAs has been frequently found in many pathophysiological processes, including cancer, underlying their crucial role in human diseases and considering them as potential diagnostic biomarkers for cancer [20,21].

We, recently, demonstrated the deregulation of lncRNA expression in PTC, and that the restoration of the downregulated *MPPED2-AS1* (*RP5-1024C24.1*) reduced cell proliferation and migration in thyroid carcinoma cell lines by modulating the PTEN/Akt pathway [22]. Then, we aimed to analyze the role of lncRNAs also in ATC.

In this study, the analysis of the lncRNAs expression in ATC and NT tissues has allowed the identification of up- and downregulated lncRNAs in ATC samples. We focused our attention on the lncRNA *Prader Willi/Angelman region RNA5* (*PAR5*) significantly and constantly decreased in ATC, but not in PTC tissues. Interestingly, the restoration of *PAR5* reduces proliferation and migration rates of two ATC-derived cell lines. Finally, we reported that *PAR5* directly interacts with EZH2 in thyroid cancer cells, reducing its binding on the *E-cadherin* promoter, then relieving E-cadherin from the negative control by EZH2 in *PAR5*-overexpressing ATC cell lines. These results suggest that *PAR5* exerts its tumor suppressor action by impairing the oncogenic activity of EZH2.

## 2. Results

### 2.1. Deregulation of lncRNAs Expression in ATC

**Figure 1:**
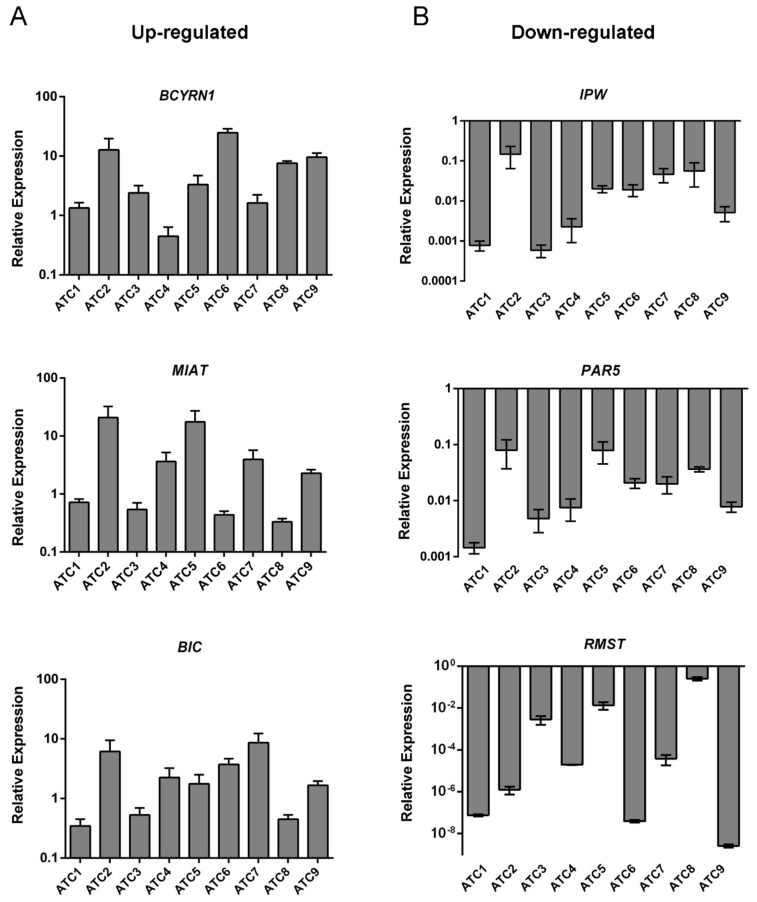
Expression analysis of lncRNAs deregulated in ATC samples. qRT-PCR analysis was carried out to evaluate the expression levels of three upregulated (**A**) and three downregulated (**B**) lncRNAs from the microarray analysis. Results are reported as 2^−ΔΔCt^ values ± standard deviation (SD) compared to the mean of five NT samples, set equal to 1.

We analyzed the lncRNA expression profile of nine human ATC tissues with respect to five NT tissues through a single channel Agilent array. This analysis revealed 47 lncRNAs aberrantly expressed in ATC (fold change >1.1 and <−1.1, *p* < 0.05, FDR ≤ 0.1), including 19 up- and 28 downregulated lncRNAs (Table S1) in comparison with NT tissues. The 10 most up- and downregulated lncRNAs, in terms of fold change are given in Table 1. The fold change and *p*-value presented for each lncRNA are the ones of the probe with the highest absolute fold change. Then, to validate the microarray results, the expression of three upregulated (*MIAT*, *BCYRN1*, *BIC*) and three downregulated (*RMST*, *PAR5*, *IPW*) lncRNAs was evaluated by qRT-PCR in the same ATC samples used for the microarray analysis. As shown in Figure 1A, the lncRNAs *MIAT*, *BCYRN1*, and *BIC* were found overexpressed whereas *RMST*, *PAR5*, and *IPW* were found significantly downregulated (*p* = 0.001) in almost all analyzed ATC tissues in comparison with the mean of five NT (Figure 1B).

### 2.2. The lncRNA PAR5 Was Drastically Downregulated in ATC, but Not in PTC

**Figure 2:**
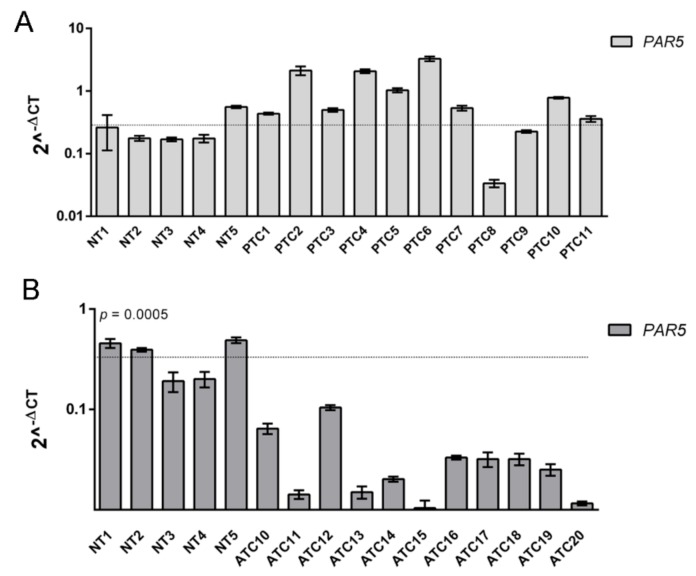
Analysis of *Prader Willi/Angelman region RNA5* (*PAR5*) expression in thyroid cancer. (**A**) qRT-PCR analysis was performed in 11 papillary thyroid carcinomas (PTC) and (**B**) an additional 11 ATC samples. *t*-test: *p* = 0.0005 (ATC vs. NT) to evaluate the expression levels of *PAR5*. The dashed line represents the average relative expression levels of five NT samples. Data are reported as 2^−ΔCt^ values ± SD.

Subsequently, our attention was focused on the lncRNA *PAR5* since it showed a drastic downregulation in all the analyzed ATC samples. Moreover, it has been reported that *PAR5* was able to directly bind EZH2 [23] that, interestingly, was found overexpressed in ATC samples, but not in the differentiated thyroid carcinomas [9]. Consequently, *PAR5* expression levels were further assessed by qRT-PCR in 11 differentiated PTC and additional 11 undifferentiated ATC samples. Intriguingly, no significant expression changes were detected in the PTC samples when compared to NT tissues (Figure 2A), while *PAR5* expression levels were drastically and significantly downregulated in all ATC samples in comparison with NT ones (*p* = 0.0005) (Figure 2B), clearly indicating that *PAR5* downregulation is strictly related to ATC progression.

### 2.3. PAR5 Overexpression Reduces Cell Proliferation of Thyroid Cancer Cell Lines

**Figure 3:**
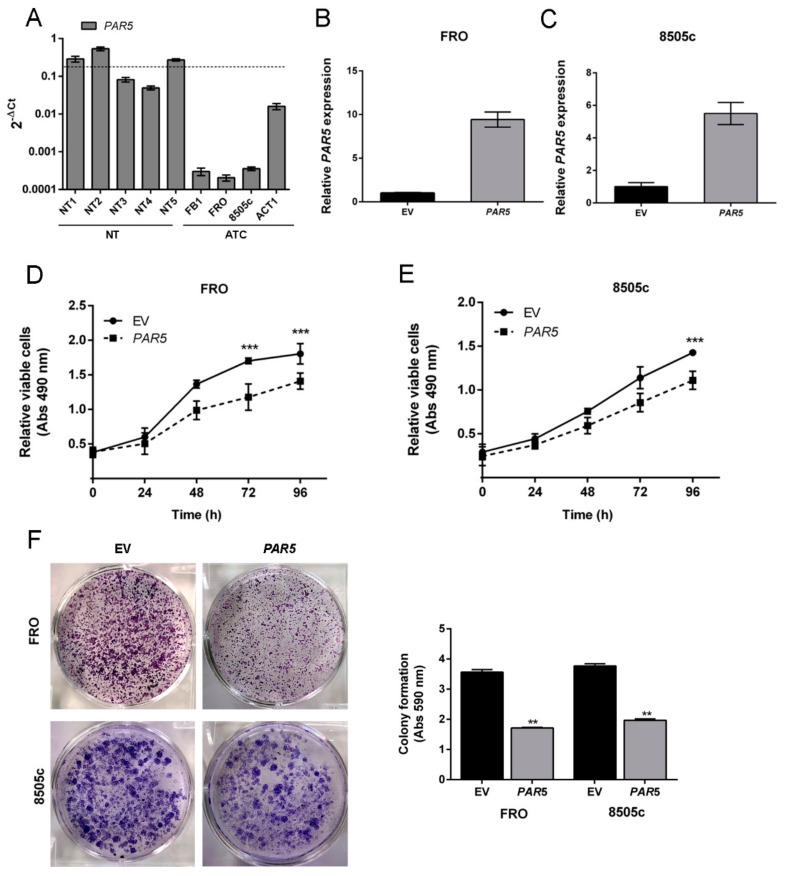
*PAR5* reduces cell proliferation of thyroid cancer cell lines. (**A**) qRT-PCR analysis of *PAR5* levels in a set of human anaplastic thyroid cell lines, including FB1, FRO, 8505c, and ACT1. Data are reported as 2^−ΔCt^ values ± SD. (**B**,**C**) qRT-PCR analysis performed on FRO and 8505c cell lines stably expressing *PAR5* or carrying the corresponding empty vector (EV). Data were compared to EV, set equal to 1, and reported as 2^−ΔΔCt^ values ± SD. (**D**,**E**) Cell growth analysis of FRO and 8505c cells stably expressing *PAR5* or carrying the corresponding EV. Cell number was evaluated at 24, 48, 72, and 96 h after seeding. Values were obtained from three independent experiments. Data are reported as mean ± SD. 2-way ANOVA test (Bonferroni post-test: *PAR5* vs. EV, 96 and 72 h, ***, *p* < 0.001 in FRO cell line, *PAR5* vs. EV, 96 h, ***, *p* < 0.001 in 8505c cell line). (**F**) A representative image of colony formation assays in FRO and 8505c cells transiently transfected with *PAR5* or the corresponding EV is reported in the left panel. Cells were stained with crystal violet after 3 weeks of selection with G418. Quantitation of colony assay was performed by dissolving crystal violet solution in 0.1% SDS and measuring the absorbance at 590 nm. The results are reported as a mean of three independent experiments (right panel). *t*-test: **, *p* < 0.01(*PAR5* vs. EV, in FRO and 8505c cells).

To better define the role of *PAR5* in thyroid cancer progression we set up functional assays to evaluate its effects on cell proliferation. First, *PAR5* levels were evaluated in four ATC-derived cell lines (FB-1, FRO, 8505c, ACT1) and, as expected, its expression was found to be extremely lower with respect to five NT used as control (Figure 3A). Then, *PAR5* expression was restored in FRO and 8505c cells by transfecting them with a *PAR5-*expressing vector (pCMV-*PAR5)*. As shown in Figure 3B,C, a great increase of *PAR5* was detected in both FRO and 8505c cells transfected with pCMV-*PAR5*, by qRT-PCR, but not in those transfected with the empty vector (EV). 

Then, the proliferation rate of *PAR5*-overexpressing ATC cells was evaluated. A significant delay of growth in the FRO-*PAR5* (*PAR5* vs. EV at 72 h and 96 h, *p* < 0.001) and 8505c-*PAR5* (*PAR5* vs. EV at 96 h, *p* < 0.001) cells was observed when compared with the corresponding EV-transfected cells (Figure 3D,E). Subsequently, to corroborate this result a colony formation assay was performed on FRO and 8505c cells transfected with pCMV-*PAR5* or EV used as control. As shown in Figure 3F, the colony number of both *PAR5*-transfected ATC cells was significantly lower in comparison with the EV control-transfected cells, confirming that *PAR5* slows down cell growth.

### 2.4. Restoration of PAR5 Expression Decreases Migratory Ability of Thyroid Cancer Cells

**Figure 4:**
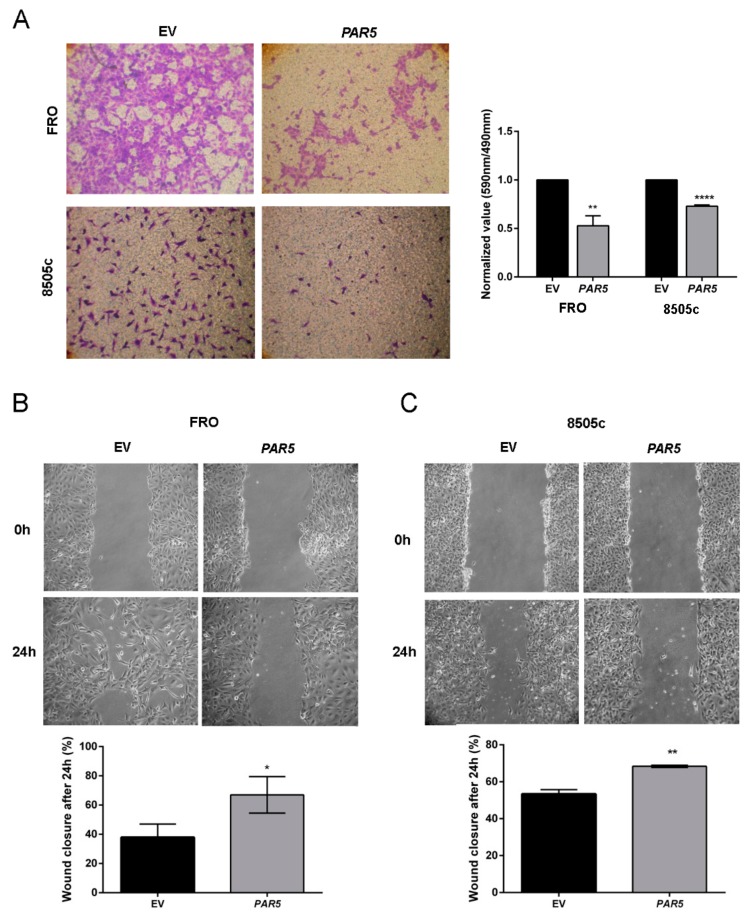
*PAR5* suppresses cell migration of ATC cell lines. (**A**) Representative images of migration assay performed in FRO and 8505c cells stably transfected with *PAR5* or the corresponding EV (left panel). Magnification 40×. Migrating cells were fixed with crystal violet solution. After elution from transwell, it was quantified by reading its absorbance at 590 nm. Crystal violet absorbance values were divided by corresponding cell titer values. In the right panel data obtained from three independent experiments carried out in FRO and 8505c cells are shown. Values are reported as mean value ± SD, compared to the EV, set equal to 1. Unpaired *t*-test: **, *p* < 0.01 (*PAR5* vs. EV, in FRO cells), ****, *p <* 0.0001 (*PAR5* vs. EV, in 8505c cells). (**B**,**C**) A representative image of scratch wound-healing assay performed in FRO and 8505c cells stably carrying *PAR5* or the corresponding EV is reported in the upper panel. *t* = 0 h and *t* = 24 h post wounding. Magnification 40×. In the lower panel is reported the percentages of closures of the wound after 24 h in FRO and 8505c cells stably expressing *PAR5* or carrying the corresponding EV. Data are presented as the average of three experiments. *, *p* < 0.05 in FRO cell line, *PAR5* vs. EV, after 24 h, **, *p* < 0.01 in 8505c cell line after 24 h.

To further evaluate the consequence of *PAR5* expression on cancer progression we analyzed the migratory ability of the *PAR5*-overexpressing ATC cells. Transwell migration assays revealed that the restored *PAR5* expression significantly reduced migration ability of about 50% (*p* < 0.01) and 30% (*p* < 0.0001) in FRO and 8505c cancer cells, respectively (Figure 4A). To deeper investigate the lncRNA influence on cell migration ability, scratch wound-healing assay was carried out in *PAR5*-overexpressing ATC cells. As shown in Figure 4B, FRO-EV cells were able to close the wound after 24 h, but this effect was not achieved by FRO-*PAR5* cells at the same time. Similar results were obtained in 8505c-*PAR5* cells, in which a significant inhibition of migration ability was detected after 24 h (Figure 4C). These results clearly evidence the role played by *PAR5* reduction in the contribution to ATC development.

### 2.5. PAR5 Negatively Modulates EZH2 in Thyroid Cancer Cells

**Figure 5:**
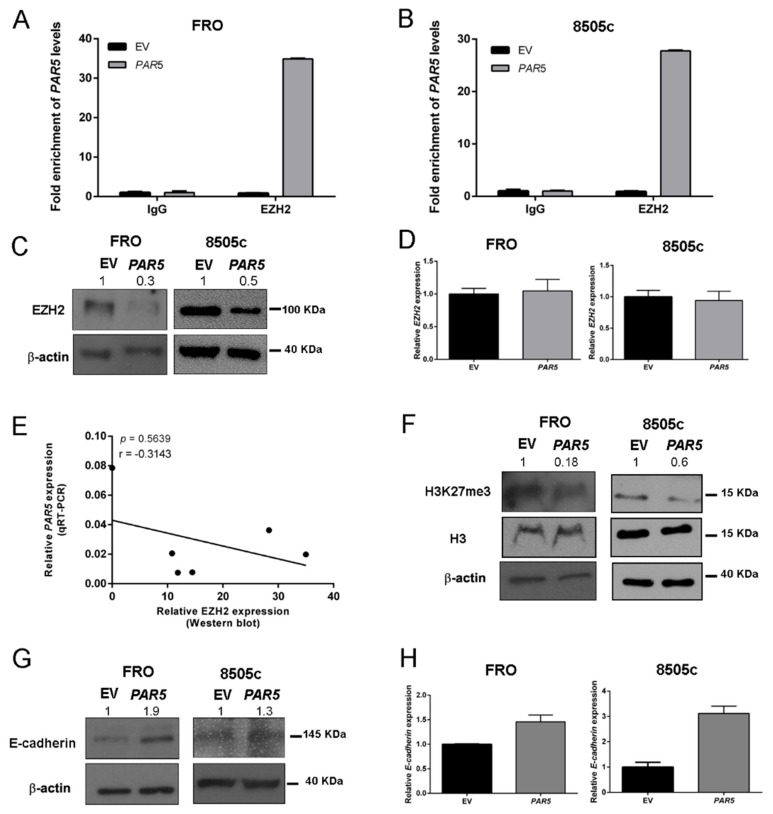
*PAR5* interacts with Enhancer of Zeste Homolog 2 (EZH2) modulating E-cadherin expression. (**A**,**B**) RNA immunoprecipitation (RIP) assay was performed on extracts obtained from FRO and 8505c cells transfected with pCMV-*PAR5* or the EV, using an anti-EZH2 antibody or a pre-immune (IgG) serum, as control. qRT-PCR was performed on the samples to assess fold enrichment of *PAR5* precipitated by the anti-EZH2 antibody relative to IgG control, set equal to 1. (**C**) Western blot analysis of EZH2 expression in FRO and 8505c stably expressing *PAR5* or carrying the corresponding EV. β-actin was used to normalize the amount of loaded protein. Densitometric analysis was performed by using ImageJ software to analyse protein expression compared to the EV, set equal to 1. (**D**) qRT-PCR analysis to evaluate the expression of *EZH2* after *PAR5* transfection. Data were reported as relative expression ± SD and were compared to the EV, set equal to 1. (**E**) Correlation scatter plot (Spearman’s Rank) between qRT-PCR levels of *PAR5* and Western blot levels of EZH2 analyzed in six ATC samples (*r* = −0.3143; *p* = 0.5639). (**F**,**G**) Western blot analysis of H3K27me3 and E-cadherin expression in FRO and 8505c stably expressing *PAR5* or carrying the corresponding EV. H3 and β-actin were used to normalize the amount of loaded protein. Densitometric analysis was performed by using ImageJ software to analyze protein expression compared to the EV, set equal to 1. (**H**) qRT-PCR analysis to evaluate the expression of *E-cadherin* after *PAR5* transfection. Data were reported as relative expression ± SD and compared to the EV, set equal to 1.

Since it has been reported that *PAR5* suppresses cell growth and migration by direct binding EZH2 in glioma cells, regulating the expression of its targets [23], we performed RNA immunoprecipitation (RIP) assays using specific EZH2 antibody in FRO and 8505c cells in order to confirm that the same mechanism occurs also in thyroid cancer system. As expected, we found an enrichment of *PAR5* in EZH2 immunoprecipitated lysates when compared to the IgG controls in both cell lines (Figure 5A,B), confirming that the interaction between *PAR5* and EZH2 also occurs in ATC cells. To further examine this mechanism, we assessed EZH2 levels after *PAR5* overexpression in FRO and 8505c cells by Western blot and qRT-PCR analyses. The obtained results showed that *PAR5* was able to reduce EZH2 protein levels in both *PAR5*-overexpressing ATC cells (Figure 5C), but no decrease of *EZH2* mRNA levels were found by qRT-PCR in FRO-*PAR5* and 8505-*PAR5* cells (Figure 5E), meaning that *PAR5* does not affect *EZH2* expression at transcriptional level. In fact, despite the higher *EZH2* mRNA levels found in the same ATC tissues by qRT-PCR, no correlation between *EZH2* mRNA and *PAR5* levels was detected (Supplementary Materials, Figure S1A). Subsequently, increased EZH2 protein levels in ATC tissues, that inversely correlate with *PAR5* RNA expression (Figure 5E), were found by Western blot analysis (Supplementary Materials, Figure S1B), suggesting that *PAR5* influences EZH2 protein expression. Additionally, the decrease of H3K27me3 levels were detected in FRO-*PAR5* and 8505-*PAR5* cells by Western blot technique (Figure 5F). Intriguingly, the reduction of EZH2 protein levels was associated with the upregulation of both mRNA and protein levels of its target gene *E-cadherin*, a key marker of epithelial-mesenchymal transition (EMT) (Figure 5G,H). 

**Figure 6:**
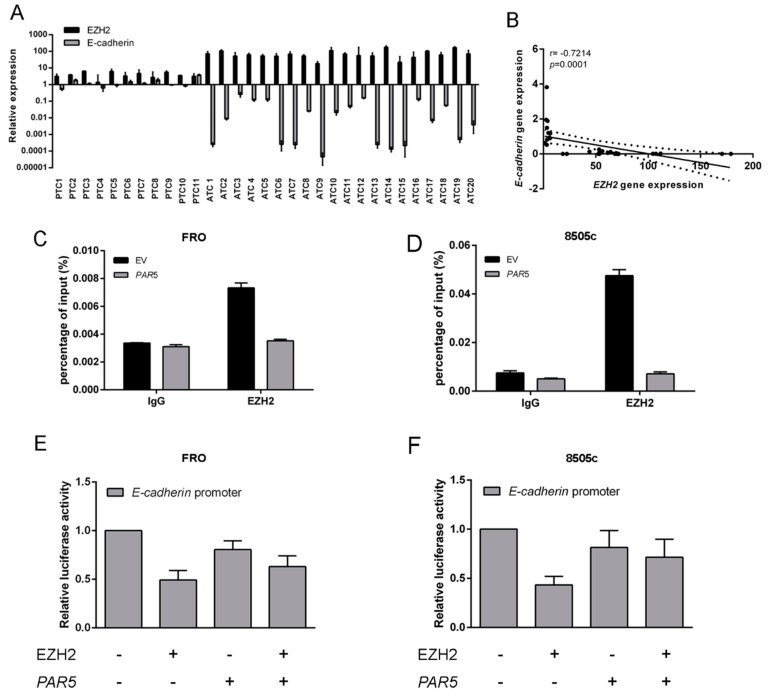
*PAR5* interacts with EZH2 reducing its binding to E-cadherin promoter. (**A**) qRT-PCR analysis was performed in 11 PTC and 20 ATC samples to evaluate the expression levels of *EZH2* and *E-cadherin.* (**B**) Correlation scatter plot (Spearman’s Rank) between *EZH2* and *E-cadherin* mRNA levels analyzed in all patient’s carcinoma samples (*r* = −0.7214; *p* = 0.0001). (**C**,**D**) Chromatin immunoprecipitation (ChIP) assay was carried out in FRO and 8505c cells transiently transfected with *PAR5* or the corresponding EV. ATC cell lines were then crosslinked, sonicated, and subjected to pre-clearing. The chromatin was immunoprecipitated using antibodies against EZH2. IgG were used as negative control. The immunoprecipitated chromatin was analyzed by qPCR assay with primers specific for the *E-cadherin* promoter. (**E**,**F**) Luciferase assays performed in ATC cells transfected with the *E-cadherin*-luc vector and with single or both vectors expressing EZH2 or *PAR5*. The total amount of the transfected DNA was balanced with the EV. Relative *E-cadherin* luciferase activity was compared to that observed in cells transfected with the only EV, assuming that the control is equal to 1. Values are the mean of three independent experiments ± SD.

Subsequently, *EZH2* and *E-cadherin* expression levels were assessed in human thyroid cancers of different histotypes by qRT-PCR. The results confirmed the specific overexpression of *EZH2* in the undifferentiated ATC tissues, while no increase was found in PTC samples (Figure 6A). Conversely a marked downregulation of *E-cadherin* was found in ATC, whereas no changes in its expression were detected in PTC samples (Figure 6A). Additionally, as shown in Figure 6B, a significant negative correlation was observed between *EZH2* and *E-cadherin* levels (*r* = −0.7214, *p* < 0.0001), meaning that EZH2 negatively regulates the *E-cadherin* gene. Then, chromatin immunoprecipitation (ChIP) assay was performed to investigate whether *PAR5* expression could modify the binding of EZH2 on *E-cadherin* promoter. To this aim, the crosslinked DNA-protein complexes were immunoprecipitated with a specific antibody raised again EZH2 or IgG. Immunoprecipitation of chromatin was then analyzed by qRT-PCR examining a region spanning nucleotides from −300 to +40 related to the transcription start site (TSS) of the *E-cadherin* promoter. As shown in Figure 6C,D, anti-EZH2 antibodies precipitated human *E-cadherin* promoter in both EV-transfected cell lines but not in those overexpressing *PAR5*. Next, the effects of EZH2-*PAR5* interaction on the modulation of the *E-cadherin* promoter were evaluated by performing luciferase assays. To this aim, ATC cell lines were transfected with a vector encoding the luciferase gene under the control of the *E-cadherin* promoter. Then, FRO and 8505 cells were transfected with single or both vectors encoding for EZH2 protein or *PAR5* gene. As shown in Figure 6E,F, *PAR5* and EZH2 behaved in an opposite way on the modulation of *E-cadherin* promoter activity. In fact, EZH2 clearly displayed a negative transcriptional regulation on *E-cadherin* promoter. On the other hand, when both genes were co-transfected, a partial rescue of the transcriptional *E-cadherin* activity was obtained, showing comparable levels to those observed in ATC cells expressing only the *PAR5* gene. These results indicate that *PAR5* not only reduces the EZH2 protein levels, but also decreases EZH2 binding to the *E-cadherin* promoter, thereby, affecting the expression of its target genes. 

## 3. Discussion

In this study we evaluated the contribution of lncRNAs to thyroid cancer progression by examining their expression profile in nine ATC and five NT samples through microarray analysis. By this approach, we discovered that several lncRNAs were differently expressed in ATC tissues in comparison with NT, underlying once more their crucial role in thyroid carcinogenesis. We, then, confirmed the microarray results by examining the expression levels of three up- and three downregulated lncRNAs by qRT-PCR. Among these lncRNAs, we focused our attention on the downregulated *PAR5,* previously proposed as a novel tumor suppressor lncRNA. Indeed, recent studies reported a drastic *PAR5* downregulation in a specific hepatitis C virus-related hepatocellular carcinoma (HCC) and gliomas [23,24]. Interestingly, a significant inverse correlation between *PAR5* levels and the malignant phenotype of glioblastoma multiforme (GBM) was described [25] underlying the critical role of *PAR5* reduction in cancer progression. Noteworthy, it has been also reported that *PAR5* can delay malignant transformation by interacting with EZH2 protein, a catalytic subunit of PRC2, that triggers H3K27 trimethylation, thereby inducing transcriptional repression of target genes implicated in important biological processes, such as cell proliferation, stem cell pluripotency, and oncogenic transformation [26,27,28,29]. Intriguingly, a significant number of studies evidenced a strict correlation of EZH2 overexpression with patient poor outcome and high aggressiveness in several cancer types [28,30,31]. Supported by this evidence, we investigated the possible functional role of *PAR5* in thyroid carcinogenesis.

To achieve this, we first analyzed *PAR5* expression levels by qRT-PCR in a set of differentiated and undifferentiated thyroid carcinoma samples. The results of this analysis showed a strong and constant *PAR5* downregulation in ATC tissues, whereas no significant changes were observed in PTC samples, meaning that *PAR5* downregulation is associated with the highly malignant phenotype also in thyroid cancer. Next, we examined the effects of *PAR5* overexpression on cellular growth and migration capability in two ATC-derived cell lines. We found that *PAR5* overexpression was able to reduce cell proliferation and cell migration ability, thus supporting the anti-oncogenic role played by this lncRNA and, thereby, the contribution of its downregulation to ATC development.

To investigate the molecular mechanisms by which the lncRNA acts in thyroid carcinogenesis we examined the effects of *PAR5* on EZH2 expression and activity since recent studies reported EZH2 overexpression in undifferentiated thyroid carcinomas, and its capability to inhibit thyroid cell differentiation [9]. Therefore, by performing RIP assays we demonstrated that *PAR5* directly binds EZH2. Moreover, we observed that *PAR5* restoration reduced EZH2 protein and H3K27me3 levels in absence of any change of *EZH2* mRNA abundance, suggesting that this lncRNA may affect EZH2 stability by a regulatory mechanism already reported for other lncRNAs [32,33]. Consistently with these findings, we found an increased EZH2 expression in ATC tissues and, interestingly, we observed a negative correlation between EZH2 protein and *PAR5* RNA levels, strongly supporting the effects of *PAR5* on EZH2.

Since it is well known that *E-cadherin*, whose lack is a hallmark of EMT [34,35,36,37], is repressed by EZH2 through H3K27 trimethylation at its promoter [29], we analyzed E-cadherin expression in the ATC-*PAR5* overexpressing cells, and increased E-cadherin protein levels were observed. Accordingly, *PAR5* restoration reduces the EZH2 binding to *E-cadherin* promoter thus preventing the EZH2 inhibitory activity on E-cadherin expression. Therefore, these results indicate that *PAR5* exerts its tumor suppressor activity by impairing the EZH2 oncogenic role. However, further investigations are needed to better understand how *PAR5* can regulate EZH2 activity. Equally, this study does not exclude that *PAR5* exerts its tumor suppressor activity interacting with other proteins involved in cancer progression. 

Moreover, our study demonstrates that *PAR5* modulates EZH2 expression. It is worth to note that other non-coding RNA, including microRNA and circular RNA, can modulate the expression of EZH2. Indeed, it has been reported that miR-25, miR-30d, and miR26a (downregulated in ATC) target EZH2 [38,39]. Additionally, circ-PRMT5 can upregulate EZH2 via sponging miR-377-382-498 in non-small cell lung cancer (NSCLC) [40]. Intriguingly, also many lncRNAs have been described to participate in the EZH2 oncogenic regulatory network [41,42], further highlighting the complex regulation of EZH2 activity in human cancer, and reinforcing its critical role in cancer progression. 

Noteworthy, other significant deregulated lncRNAs came out from the microarray analysis. In fact, it would be worthwhile to better investigate the role in ATC development of the downregulated *rhabdomyosarcoma 2-associated transcript (RMST)*, a lncRNA able to regulate neurogenesis through the interaction with SOX2 (a critical player in pluripotent stem cell) [43]. *RMST* also exerted anti-oncogenic role in triple-negative breast cancer (TNBC) since it was found downregulated in TNBC tissues, where its restoration inhibits cell proliferation, cell cycle, invasion, and migration in TNBC cells [44]. Additionally, it has been found that this lncRNA is even able to enhance DNMT3 expression by interacting with HuR, thus leading to an aberrant DNA methylation pattern [45]. Interestingly, our preliminary results show that also *RMST* expression was decreased in ATC samples, without any change in PTC tissues. It is quite interesting to observe that lncRNAs, similarly to miRNAs [10,46], show an opposite behavior in the different subtype of thyroid carcinomas, in which their expression was found upregulated or unchanged in PTC and downregulated in the most aggressive undifferentiated subtypes, highlighting the critical role of lncRNA deregulation in the process that leads to ATC development.

In conclusion, our findings demonstrate that *PAR5* downregulation was strictly related to the undifferentiated thyroid carcinomas and that *PAR5* expression affected cell growth and migration rate by interacting with EZH2 and inhibiting its oncogenic activity.

## 4. Materials and Methods

### 4.1. Human Thyroid Tissue Samples

Normal and neoplastic human thyroid tissues were obtained by the Service d’Anatomie et Cytologie Pathologiques, Centre de Biologie Sud, Groupement Hospitalier Lyon Sud, Pierre Bénite, France. Informed consent was obtained from all patients. The activity of biological samples conservation was declared under the number DC-2011-1437 to the Ministry of Research, to the committee of people’s protection of south-east IV and to the Health Regional Agency. The activity of biological material cession was agreed upon by the Ministry of Health under the number AC-2013-1867.

### 4.2. Array Design and Data Analysis

The arrays utilize nucleic acid hybridization of a 52 nt biotin-labeled cDNA target with DNA oligonucleotide probes attached to a gel matrix. The biotin-labeled cDNA targets were prepared by a reverse transcription into first strand cDNA. Total RNA extracted from nine ATC and five NT was primed for reverse transcription by a random octamer conjugated with two biotins and a 52 nt long poly-A tail. This procedure results in an equal copy number of biotin cDNA targets to the ncRNA templates [47]. The array is a customized single-channel Agilent array (Agilent Technologies, Santa Clara, CA, USA). It contains a collection of probes (sense and antisense) for various types of non-coding RNAs (18009 probes corresponding to 1271 human pre-miRNAs, 8660 probes corresponding to 626 mouse pre-miRNAs (miRBase 21), 2745 probes corresponding to 479 ultra-conserved elements, 16,314 probes corresponding to 1283 pyknon, and 2197 probes corresponding to 97 long non-coding RNAs). Data preprocessing steps of background-correction, normalization, and summarization were performed in R version 3.5.1 (https://www.r-project.org/) using functions in Limma library (http://www.bioconductor.org/packages/devel/bioc/vignettes/limma/inst/doc/usersguide.pdf).

A threshold for positive spot selection for microarray data was calculated as the mean value of all the dark corner spots plus twice the standard deviation [48]. For class comparison we employed again Lima library, a linear model was fitted to each gene, and empirical Bayes methods were used to obtain the statistics. The statistical significance was defined as a *p*-value < 0.05 and we imposed a cut-off of functional relevance on the fold change in absolute value of 1.1.

### 4.3. Cell Culture and Transfections

Human thyroid cancer cell lines FRO and 8505c were grown in DMEM (Sigma-Aldrich, St. Louis, MO, USA) supplemented with 10% fetal bovine serum (FBS) (Euroclone, Milan, Italy), 1% L-glutamine, 1% penicillin/streptomycin (Sigma-Aldrich). Cells were maintained at 37 °C under 5% CO_2_ atmosphere. Lipofectamine 2000 (Life Technologies, Grand Island, NY, USA) reagent was used to transfect the cells according to the manufacturer’s instructions. The stable-transfected FRO and 8505c cells were selected in a medium containing 1200 and 1000 µg/mL of G418 (Life Technologies), respectively.

### 4.4. RNA Extraction and Quantitative Real-Time PCR (qRT-PCR)

Total RNA from thyroid cancer tissues and cell lines were extracted using Trizol reagent (Life Technologies). A total of 1 µg of total RNA from each sample was used to obtain double strand cDNA with the QuantiTect Reverse Transcription Kit (Qiagen, Hilden, Germany). Quantitative Real-Time PCR (qRT-PCR) was performed with the CFX96 thermocycler (Bio-Rad, Hercules, CA, USA) in 96-well plates. For each PCR reaction, 10 µL of 2× Sybr Green (Bio-Rad), 200 nM of each primer, and 20 ng of the cDNA, previously generated, were used. The oligonucleotides for qRT-PCR, encompassing exon-exon junctions, were purchased from Integrated DNA Technologies (San Diego, CA, USA) and designed with Primer-BLAST software. Relative gene expression was determined using comparative C(T) method, as described elsewhere. Peptidylprolyl isomerase A (PPIA) was used as housekeeping gene [49,50]. Detailed primer sequences are available as Supplementary Materials, Table S2.

### 4.5. Plasmids

The expression vector encoding human *PAR5* gene was generated by cloning cDNA sequence in the pCMV6-AC-GFP vector (Origene Technologies, Rockville, MD, USA) by using In-Fusion cloning method (Takara, Beijing, China) and HindIII restriction site. After cloning, the plasmid was subjected to sequencing (Eurofins Genomics, Vimodrone, Italy) and *PAR5* expression was validated by qRT-PCR analysis.

### 4.6. RNA Immunoprecipitation (RIP) Assay 

RIP experiments were performed using the Magna RIP™ RNA-Binding Protein Immunoprecipitation Kit (Millipore, Billerica, MA, USA) according to the manufacturer’s protocol. Briefly, FRO and 8505c cells were lysed in RIP lysis buffer. First, 5 µg of human anti-EZH2 antibody (#4905, Cell signaling, Danvers, MA, USA) and normal rabbit IgG (Millipore) used as negative control, were incubated with magnetic beads for 30 min. Then, 100 μL of whole lysates were incubated overnight on a rocking platform at 4 °C. The next day, samples were incubated with Proteinase K buffer at 65 °C for 30 min and then immunoprecipitated RNA was purified. Purified RNA was reverse transcribed into cDNA by using random primer with the QuantiTect Reverse Transcription Kit (Qiagen), and the presence of *PAR5* transcripts was detected by qRT-PCR. Enrichment of *PAR5* transcripts in the EZH2 immunoprecipitated lysate was calculated relative to the levels of *PAR5* transcript in the rabbit IgG control sample, set equal to 1 [51,52].

### 4.7. Chromatin Immunoprecipitation (ChIP) Assay

ChIP experiments were performed in ATC cell lines transiently transfected with *PAR5-*overexpressing vector. Briefly, 48 h after transfection, 5 × 10^6^ FRO and 8505c cells were cross-linked to fix the DNA/protein complexes using 1% formaldehyde at RT for 10 min and the reaction was then stopped by adding glycine at a final concentration of 0.125 M. Cells were lysed in 300 μL of buffer containing 10 mM EDTA, 50 mM Tris-HCl pH 8.0, 1% SDS and protease inhibitors and then sonicated three times for 30 cycles (30 sec On, 30 sec Off) at maximum settings (Bioruptor^TM^ Next Gen, Diagenode Inc., Denville, NJ, USA), obtaining fragments between 0.3 and 1.0 kb. After centrifuging samples at 14,000 rpm for 15 min at 4 °C, 3% of supernatant amount was used as control of the total chromatin obtained (input), and the remaining part of the sample was diluted 2.5-fold in IP buffer (100 mM NaCl, 2 mM EDTA pH 8.0, 20 mM Tris-HCl pH 8.0, 0.5% Triton X-100 and protease inhibitors). After 3 h of pre-clearing at 4 °C with protein A-Sepharose saturated with salmon sperm (Millipore), samples were mixed overnight at 4 °C with the EZH2 antibodies (#4905, Cell signaling) and rabbit normal IgG (sc-2027, Santa Cruz Biotechnology, Santa Cruz, CA, USA). Subsequently, the DNA-protein-antibodies complexes were immunoprecipitated with the protein A previously used and then the chromatin was released from the beads through 30 min incubation with 250 μL of 1% SDS, 0.1 M NaHCO_3_ at 37 °C and finally with 200 nM NaCl at 65 °C overnight. Subsequently, 10 μL of 0.5 mM EDTA, 20 μL of 1 M Tris-HCl pH 6.5 and 20 μg of Proteinase K were added to the reaction tube and then the complexes were incubated for 1 h at 45 °C. DNA from chromatin immunoprecipitation was purified by phenol/chloroform extraction (Life Technologies) and precipitated by adding two volumes of ethanol and 0.1 M CH_3_COONa. IgG were used as non-specific control and input DNA values were used to normalize the values from ChIP samples. The percentage of IP chromatin was calculated as 2^−ΔCt^ × 3, where ΔCt is the difference between ^Ct^input and ^Ct^IPsample, and 3 is the percentage of total sample used for the input. The relative abundance of immunoprecipitated chromatin was expressed as percentage of binding of interested promoter compared to the input. Detailed primer sequences are available as Supplementary Materials, Table S2.

### 4.8. Luciferase Assay

First, 3. × 10^5^ ATC cells were seeded in a 6-well plate, transfected with 100 ng of the *E*-cadherin-luciferase reporter gene [53] and expression vectors encoding the EZH2 and *PAR5*. Then, 24 h after transfection, cell extracts were prepared and the luciferase activity was measured by using a Lumat LB9507 luminometer (Berthold Technologies, Bad Wildbad, Germany) and the Dual-Luciferase Reporter System kit (Promega, Fitchburg, WI, USA). A vector expressing Renilla gene under the control of the cytomegalovirus (CMV) promoter was used to normalize transfection efficiency. For all transfections, the total amount of the transfected DNA was balanced with the empty vector.

### 4.9. Western Blot

Cells were homogenized in a lysis buffer containing 150 mM NaCl, 1% Triton-X-100, 50 mM HEPES, 1 mM EDTA, 1 mM EGTA, 10% glycerol, 1 μg aprotinin, 1 mM phenylmethylsulphonyl fluoride, 0.5 mM sodium orthovanadate, 20 mM sodium pyrophosphate, and a mix of protease inhibitors. Cell lysates were then subjected to SDS/PAGE and then transferred onto Immobilon-P transfer membranes (Merck Millipore, Burlington, MA, USA). Membranes were blocked with 5% non-fat milk and probed with the indicated antibodies at the appropriate dilutions: EZH2 (#3147, Cell signaling), H3K27me3 (#07449, Upstate biotechnology, Billerica, MA, USA), H3 (#ab1791, Abcam, Cambridge, UK), E-cadherin (#3195, Cell signaling), and β-actin (#A5441, Sigma-Aldrich). Membranes were incubated with horseradish peroxidase-conjugated secondary antibody (1:3000) for 60 min at RT. Signals were detected with chemiluminescent detection system (ECL) (Thermo Fisher Scientific, Waltham, MA, USA), and films were developed with a semiautomatic developing machine (Cawomat IR 2000, CAWO Photochemisches, Schrobenhausen, Germany). Original blots were shown in Supplementary Materials Figures S2 and S3. Densitometric analyses of the Western blot bands were obtained using ImageJ 1.43 software (NIH, Bethesda, MD, USA) [54].

### 4.10. Cell Migration Assay

Transwell migration assays were performed using transwell chamber (8-μm pores). Briefly, thyroid cancer cells (3 × 10^4^) were plated in the upper transwell chamber in serum-free medium. Then, 0.3 mL complete medium was added in the lower chamber as chemoattractant. After 24 h of incubation, migrated cells on the membrane of the chambers were fixed and stained with crystal violet solution (crystal violet 0.05%, methanol 20%). FRO and 8505c cells were also seeded in a 96-well plate to normalize the number of the cells. After 2 h, the absorbance at 490 nm was read using cell titer (Promega) in a microplate reader (LX800, Universal Microplate Reader, BioTek Instruments, Inc., Winooski, VT, USA). Then, crystal violet in the chamber was de-stained with PBS-0.1% SDS solution and was read at 590 nm. Results were obtained by normalizing the crystal violet values to cell titer ones.

### 4.11. Wound-Healing Assay

Anaplastic thyroid cancer cells overexpressing *PAR5* or carrying EV (as control) were grown to confluence in 60 mm plates in DMEM medium containing 10 µg/mL Mitomycin C (#M4287, Sigma-Aldrich) for 2 h to completely inhibit cell proliferation. A linear scratch was made on the monolayer using a P200 pipette tip. The cells were then washed with PBS three times, and further cultured in DMEM medium. After 24 h of incubation, the gap size of scratch was measured and recorded, and then compared with the initial gap size at 0 h. Using the ImageJ 1.43 image processing program (NIH), the wound closed area was determined at each time point from the digital images. 

### 4.12. Colony Formation Assay

FRO and 8505c cells at 80% of confluency in 6-well plates were transfected with pCMV6-AC-GFP-EV and pCMV6-AC-GFP-*PAR5*. After 48 h of transfection, cells were treated with 1200 µg/mL and 1000 µg/mL G418, respectively. Medium containing G418 was refreshed every two days and, after 3 weeks of antibody selection, cells were fixed and stained with a solution containing crystal violet. Then, the stained crystal violet was resolved with PBS-0.1% SDS and absorbance at 590 nm was determined. The data were performed in triplicate and showed as mean ± SD.

### 4.13. Growth Curve Assay

First, 5 × 10^3^ FRO and 8505c cells overexpressing *PAR5* and carrying the EV were plated in 96-well plates. Cell growth was assessed using CellTiter 96^®^ AQueous One Solution Cell Proliferation Assay (MTS) (Promega), at 0, 24, 48, 72, and 96 h, as previously reported [55]. Measures were performed at 490 nm using a microplate reader (Lx800, BioTeK Instruments).

### 4.14. Statistical Analysis

All data were reported as mean ± standard deviation (SD). Results were analyzed using Student’s *t*-test, Mann–Whitney’s test and ANOVA test, when required. The correlations were evaluated through non-parametric Spearman’s Rank correlation coefficient with 95% confidence interval. Statistical analyses were performed using GraphPad Prism software 6.0 and the difference was considered significant when *p* < 0.05.

## 5. Conclusions

In conclusion, the results reported here strongly support the key role played by the lncRNA *PAR5* during thyroid carcinogenesis. Indeed, we showed that the reduction of *PAR5* was strictly related to the most aggressive ATC phenotype, where its restoration is important to inhibit the main feature of thyroid cancer cell lines. Additionally, we showed that *PAR5* acted by impairing the activity of EZH2, relieving the downstream target gene *E-cadherin* from EZH2 suppression. Our findings thus demonstrated the anti-oncogenic role of *PAR5* in thyroid carcinogenesis, suggesting a new mechanism of action by which the lncRNAs impair cancer progression.

## Figures and Tables

**Table 1:** Representative table of deregulated long non-coding RNAs (lncRNAs) in anaplastic thyroid carcinoma (ATC) with respect to normal thyroid (NT) tissues.

Upregulated lncRNAs (ATCs vs. NT)				
Gene Symbol	Seqname	Fold Change	*p*-Value	Chr
*MIAT*	NR_033319.2	4.015641156	0.027858355	22
*BCYRN1*	NR_001568.1	3.70866242	0.010905362	2
*BIC*	NR_001458.3	2.935717391	0.038708669	21
*CDKN2B-AS1*	NR_003529.3	2.001715424	0.031736686	21
*DQ866763*	DQ866763.1	1.466828568	0.024543475	13
*AJ010230*	NR_002727.2	1.409412721	0.03249904	22
*HSU62668*	NR_120534.1	1.335028913	0.001106524	11
*AY072610*	AY072610.1	1.315029623	0.012971808	4
*DQ866756*	DQ866756.1	1.251182892	0.040449836	7
*HOTAIR*	NR_047517.1	1.179044375	0.007674458	12
**Downregulated lncRNAs (ATCs vs. NT)**				
**Gene Symbol**	**Seqname**	**Fold Change**	***p*-Value**	**Chr**
*RMST*	NR_024037.1	−129.3251308	1.59 × 10^−5^	22
*PAR5*	NR_022008.1	−14.77494623	2.31 × 10^−6^	15
*IPW*	NR_023915.1	−6.082183153	6.26 × 10^−7^	15
*DQ866762*	DQ866762.1	−3.914930294	2.16 × 10^−5^	13
*MALAT1*	NR_002819.3	−2.762370686	0.004884614	11
*EMX2OS*	NR_002791.2	−2.743483886	0.000478037	10
*AY166681*	AY166681.1	−2.614571419	0.00012898	6
*XIST*	NR_001564.2	−2.513889473	0.005014223	X
*AK057701*	AK057701.1	−2.214342729	0.003083716	X
*D43770*	D43770.1	−2.192780556	0.009570036	17

## Supplementary Materials

The following are available online at https://www.mdpi.com/2072-6694/12/1/235/s1, Figure S1: Analysis of EZH2 expression in ATC samples, Figure S2: Originals blots for Western blot relative to β-actin, H3K27me3, H3, E-cadherin, and EZH2 in FRO and 8505c cell lines, Figure S3: Originals blots for Western blot relative to β-actin and EZH2 in ATC and NT samples, Table S1: Whole list of lncRNAs deregulated in ATC vs. NT, Table S2: List of primers used for the following analysis.

## References

[1] Fagin J.A., Wells S.A.J. (2016). Biologic and Clinical Perspectives on Thyroid Cancer. N. Engl. J. Med..

[2] Nikiforov Y.E., Nikiforova M.N. (2011). Molecular genetics and diagnosis of thyroid cancer. Nat. Rev. Endocrinol..

[3] Kebebew E., Greenspan F.S., Clark O.H., Woeber K.A., McMillan A. (2005). Anaplastic thyroid carcinoma. Treatment outcome and prognostic factors. Cancer.

[4] Fagin J.A., Matsuo K., Karmakar A., Chen D.L., Tang S.H., Koeffler H.P. (1993). High prevalence of mutations of the p53 gene in poorly differentiated human thyroid carcinomas. J. Clin. Investig..

[5] Donghi R., Longoni A., Pilotti S., Michieli P., Della Porta G., Pierotti M.A. (1993). Gene p53 mutations are restricted to poorly differentiated and undifferentiated carcinomas of the thyroid gland. J. Clin. Investig..

[6] Landa I., Ibrahimpasic T., Boucai L., Sinha R., Knauf J.A., Shah R.H., Dogan S., Ricarte-Filho J.C., Krishnamoorthy G.P., Xu B. (2016). Genomic and transcriptomic hallmarks of poorly differentiated and anaplastic thyroid cancers. J. Clin. Investig..

[7] Shi X., Liu R., Qu S., Zhu G., Bishop J., Liu X., Sun H., Shan Z., Wang E., Luo Y. (2015). Association of TERT promoter mutation 1,295,228 C>T with BRAF V600E mutation, older patient age, and distant metastasis in anaplastic thyroid cancer. J. Clin. Endocrinol. Metab..

[8] Liu T., Wang N., Cao J., Sofiadis A., Dinets A., Zedenius J., Larsson C., Xu D. (2014). The age-and shorter telomere-dependent TERT promoter mutation in follicular thyroid cell-derived carcinomas. Oncogene.

[9] Borbone E., Troncone G., Ferraro A., Jasencakova Z., Stojic L., Esposito F., Hornig N., Fusco A., Orlando V. (2011). Enhancer of zeste homolog 2 overexpression has a role in the development of anaplastic thyroid carcinomas. J. Clin. Endocrinol. Metab..

[10] Visone R., Pallante P., Vecchione A., Cirombella R., Ferracin M., Ferraro A., Volinia S., Coluzzi S., Leone V., Borbone E. (2007). Specific microRNAs are downregulated in human thyroid anaplastic carcinomas. Oncogene.

[11] Kim E.D., Sung S. (2012). Long noncoding RNA: Unveiling hidden layer of gene regulatory networks. Trends Plant Sci..

[12] Kapranov P., Cheng J., Dike S., Nix D.A., Duttagupta R., Willingham A.T., Stadler P.F., Hertel J., Hackermuller J., Hofacker I.L. (2007). RNA maps reveal new RNA classes and a possible function for pervasive transcription. Science.

[13] Ulitsky I., Bartel D.P. (2013). lincRNAs: Genomics, evolution, and mechanisms. Cell.

[14] Gupta R.A., Shah N., Wang K.C., Kim J., Horlings H.M., Wong D.J., Tsai M.C., Hung T., Argani P., Rinn J.L. (2010). Long non-coding RNA HOTAIR reprograms chromatin state to promote cancer metastasis. Nature.

[15] Tsai M.C., Manor O., Wan Y., Mosammaparast N., Wang J.K., Lan F., Shi Y., Segal E., Chang H.Y. (2010). Long noncoding RNA as modular scaffold of histone modification complexes. Science.

[16] Wapinski O., Chang H.Y. (2011). Long noncoding RNAs and human disease. Trends Cell Biol..

[17] Wang K.C., Chang H.Y. (2011). Molecular mechanisms of long noncoding RNAs. Mol. Cell.

[18] Yang F., Huo X.S., Yuan S.X., Zhang L., Zhou W.P., Wang F., Sun S.H. (2013). Repression of the long noncoding RNA-LET by histone deacetylase 3 contributes to hypoxia-mediated metastasis. Mol. Cell.

[19] Pellecchia S., Sepe R., Federico A., Cuomo M., Credendino S.C., Pisapia P., Bellevicine C., Nicolau-Neto P., Severo Ramundo M., Crescenzi E. (2019). The Metallophosphoesterase-Domain-Containing Protein 2 (MPPED2) Gene Acts as Tumor Suppressor in Breast Cancer. Cancers (Basel).

[20] Martens-Uzunova E.S., Bottcher R., Croce C.M., Jenster G., Visakorpi T., Calin G.A. (2014). Long noncoding RNA in prostate, bladder, and kidney cancer. Eur. Urol..

[21] D’Angelo D., Mussnich P., Sepe R., Raia M., Del Vecchio L., Cappabianca P., Pellecchia S., Petrosino S., Saggio S., Solari D. (2019). RPSAP52 lncRNA is overexpressed in pituitary tumors and promotes cell proliferation by acting as miRNA sponge for HMGA proteins. J. Mol. Med..

[22] Sepe R., Pellecchia S., Serra P., D’Angelo D., Federico A., Raia M., Cortez Cardoso Penha R., Decaussin-Petrucci M., Del Vecchio L., Fusco A. (2018). The Long Non-Coding RNA RP5-1024C24.1 and Its Associated-Gene MPPED2 Are Down-Regulated in Human Thyroid Neoplasias and Act as Tumour Suppressors. Cancers (Basel).

[23] Wang X.P., Shan C., Deng X.L., Li L.Y., Ma W. (2017). Long non-coding RNA PAR5 inhibits the proliferation and progression of glioma through interaction with EZH2. Oncol. Rep..

[24] Zhang Q., Matsuura K., Kleiner D.E., Zamboni F., Alter H.J., Farci P. (2016). Analysis of long noncoding RNA expression in hepatocellular carcinoma of different viral etiology. J. Transl. Med..

[25] Zhang X.Q., Sun S., Lam K.F., Kiang K.M., Pu J.K., Ho A.S., Lui W.M., Fung C.F., Wong T.S., Leung G.K. (2013). A long non-coding RNA signature in glioblastoma multiforme predicts survival. Neurobiol. Dis..

[26] Simon J.A., Lange C.A. (2008). Roles of the EZH2 histone methyltransferase in cancer epigenetics. Mutat. Res..

[27] Bracken A.P., Pasini D., Capra M., Prosperini E., Colli E., Helin K. (2003). EZH2 is downstream of the pRB-E2F pathway, essential for proliferation and amplified in cancer. EMBO J..

[28] Kleer C.G., Cao Q., Varambally S., Shen R., Ota I., Tomlins S.A., Ghosh D., Sewalt R.G., Otte A.P., Hayes D.F. (2003). EZH2 is a marker of aggressive breast cancer and promotes neoplastic transformation of breast epithelial cells. Proc. Natl. Acad. Sci. USA.

[29] Cao R., Zhang Y. (2004). The functions of E(Z)/EZH2-mediated methylation of lysine 27 in histone H3. Curr. Opin. Genet. Dev..

[30] Varambally S., Dhanasekaran S.M., Zhou M., Barrette T.R., Kumar-Sinha C., Sanda M.G., Ghosh D., Pienta K.J., Sewalt R.G., Otte A.P. (2002). The polycomb group protein EZH2 is involved in progression of prostate cancer. Nature.

[31] Bachmann I.M., Halvorsen O.J., Collett K., Stefansson I.M., Straume O., Haukaas S.A., Salvesen H.B., Otte A.P., Akslen L.A. (2006). EZH2 expression is associated with high proliferation rate and aggressive tumor subgroups in cutaneous melanoma and cancers of the endometrium, prostate, and breast. J. Clin. Oncol..

[32] Li Z., Hou P., Fan D., Dong M., Ma M., Li H., Yao R., Li Y., Wang G., Geng P. (2017). The degradation of EZH2 mediated by lncRNA ANCR attenuated the invasion and metastasis of breast cancer. Cell Death Differ..

[33] Jin L., Cai Q., Wang S., Mondal T., Wang J., Quan Z. (2018). Long noncoding RNA MEG3 regulates LATS2 by promoting the ubiquitination of EZH2 and inhibits proliferation and invasion in gallbladder cancer. Cell Death Dis..

[34] Umbas R., Isaacs W.B., Bringuier P.P., Schaafsma H.E., Karthaus H.F., Oosterhof G.O., Debruyne F.M., Schalken J.A. (1994). Decreased E-cadherin expression is associated with poor prognosis in patients with prostate cancer. Cancer Res..

[35] Mayer B., Johnson J.P., Leitl F., Jauch K.W., Heiss M.M., Schildberg F.W., Birchmeier W., Funke I. (1993). E-cadherin expression in primary and metastatic gastric cancer: Down-regulation correlates with cellular dedifferentiation and glandular disintegration. Cancer Res..

[36] Frixen U.H., Nagamine Y. (1993). Stimulation of urokinase-type plasminogen activator expression by blockage of E-cadherin-dependent cell-cell adhesion. Cancer Res..

[37] Pierceall W.E., Woodard A.S., Morrow J.S., Rimm D., Fearon E.R. (1995). Frequent alterations in E-cadherin and alpha- and beta-catenin expression in human breast cancer cell lines. Oncogene.

[38] Esposito F., Tornincasa M., Pallante P., Federico A., Borbone E., Pierantoni G.M., Fusco A. (2012). Down-regulation of the miR-25 and miR-30d contributes to the development of anaplastic thyroid carcinoma targeting the polycomb protein EZH2. J. Clin. Endocrinol. Metab..

[39] Zhuang C., Wang P., Huang D., Xu L., Wang X., Wang L., Hu L. (2016). A double-negative feedback loop between EZH2 and miR-26a regulates tumor cell growth in hepatocellular carcinoma. Int. J. Oncol..

[40] Wang Y., Li Y., He H., Wang F. (2019). Circular RNA circ-PRMT5 facilitates non-small cell lung cancer proliferation through upregulating EZH2 via sponging miR-377/382/498. Gene.

[41] Pan M.Z., Song Y.L., Gao F. (2019). MiR-605-3p inhibits malignant progression of prostate cancer by up-regulating EZH2. Eur. Rev. Med. Pharmacol. Sci..

[42] Li B., Xie D., Zhang H. (2019). MicroRNA-101-3p advances cisplatin sensitivity in bladder urothelial carcinoma through targeted silencing EZH2. J. Cancer.

[43] Ng S.Y., Bogu G.K., Soh B.S., Stanton L.W. (2017). The long noncoding RNA RMST interacts with SOX2 to regulate neurogenesis. Mol. Cell.

[44] Wang L., Liu D., Wu X., Zeng Y., Li L., Hou Y., Li W., Liu Z. (2017). Long non-coding RNA (LncRNA) RMST in triple-negative breast cancer (TNBC): Expression analysis and biological roles research. J. Cell. Physiol..

[45] Peng W.X., Koirala P., Zhang W., Ni C., Wang Z., Yang L., Mo Y.Y. (2019). lncRNA RMST Enhances DNMT3 Expression through Interaction with HuR. Mol. Ther..

[46] Pallante P., Visone R., Croce C.M., Fusco A. (2010). Deregulation of microRNA expression in follicular-cell-derived human thyroid carcinomas. Endocr. Relat. Cancer.

[47] Dragomir M.P., Tudor S., Okubo K., Shimizu M., Chen M., Giza D.E., He W.R., Ivan C., Calin G.A., Vasilescu C. (2019). The non-coding RNome after splenectomy. J. Cell Mol. Med..

[48] Vallee M., Gravel C., Palin M.F., Reghenas H., Stothard P., Wishart D.S., Sirard M.A. (2005). Identification of novel and known oocyte-specific genes using complementary DNA subtraction and microarray analysis in three different species. Biol. Reprod..

[49] Forzati F., De Martino M., Esposito F., Sepe R., Pellecchia S., Malapelle U., Pellino G., Arra C., Fusco A. (2017). miR-155 is positively regulated by CBX7 in mouse embryonic fibroblasts and colon carcinomas, and targets the KRAS oncogene. BMC Cancer.

[50] Palumbo A.J., Da Costa N.M., De Martino M., Sepe R., Pellecchia S., de Sousa V.P., Nicolau Neto P., Kruel C.D., Bergman A., Nasciutti L.E. (2016). UBE2C is overexpressed in ESCC tissues and its abrogation attenuates the malignant phenotype of ESCC cell lines. Oncotarget.

[51] Fu Y., Li C., Luo Y., Li L., Liu J., Gui R. (2018). Silencing of Long Non-coding RNA MIAT Sensitizes Lung Cancer Cells to Gefitinib by Epigenetically Regulating miR-34a. Front. Pharmacol..

[52] Wu J.H., Tang J.M., Li J., Li X.W. (2018). Upregulation of SOX2-activated lncRNA ANRIL promotes nasopharyngeal carcinoma cell growth. Sci. Rep..

[53] Federico A., Pallante P., Bianco M., Ferraro A., Esposito F., Monti M., Cozzolino M., Keller S., Fedele M., Leone V. (2009). Chromobox protein homologue 7 protein, with decreased expression in human carcinomas, positively regulates E-cadherin expression by interacting with the histone deacetylase 2 protein. Cancer Res..

[54] Cacciola N.A., Sepe R., Forzati F., Federico A., Pellecchia S., Malapelle U., De Stefano A., Rocco D., Fusco A., Pallante P. (2015). Restoration of CBX7 expression increases the susceptibility of human lung carcinoma cells to irinotecan treatment. Naunyn. Schmiedebergs Arch. Pharmacol..

[55] Penha R.C.C., Sepe R., De Martino M., Esposito F., Pellecchia S., Raia M., Del Vecchio L., Decaussin-Petrucci M., De Vita G., Pinto L.F.R. (2017). Role of Dicer1 in thyroid cell proliferation and differentiation. Cell Cycle.

