# Supplementary material for: The Long Non-Coding RNA Prader Willi/Angelman Region RNA5 (PAR5) Is Downregulated in Anaplastic Thyroid Carcinomas Where It Acts as a Tumor Suppressor by Reducing EZH2 Activity
Source: Cancers (Basel). 2020 Jan 17;12(1):235. doi: 10.3390/cancers12010235 (PMC7017000; doi:10.3390/cancers12010235)
Supplement: Supplementary file 1 [file cancers-12-00235-s001.zip › cancers-678326-suppl-proof.doc]

**SUPPLEMENTARY MATERIAL**

**SUPPLEMENTARY FIGURES**

A

B

**EZH2**

**β-actin**

**Figure S1.** Analysis of EZH2 expression in ATC samples. **(A)** qRT-PCR was performed in 9 ATC tissues to analyze EZH2 mRNA expression levels. Results are reported as 2^−ΔΔCt^ values ± standard deviation (SD) compared to the mean of 5 NT samples, set equal to 1. **(B)** Western blot analysis was assessed to evaluate EZH2 protein levels in 3 NT and 6 ATC samples. β-actin was used to normalize the amount of loaded protein. Original blots were shown in Figure S3.

**SUPPLEMENTARY TABLES**

**Table S2.** List of primers used for the following analysis:

**Primers used for qRT-PCR experiment**

| **Primer name** | **Sequence 5'-3'** |
| --- | --- |
| *MIAT Forward* | GGACTGGTTCCATTCCATCAAAGG |
| *MIAT Reverse* | CCCTGGGGGTAATGACATGG |
| *BCYRN1 Forward* | CTGGGCAATATAGCGAGAC |
| *BCYRN1 Reverse* | TGCTTTGAGGGAAGTTACG |
| *BIC Forward* | ACGGTTGTGCGAGCAGAGAATCTA |
| *BIC Reverse* | CTCATCTAAGCCTCACAACAACCT |
| *RMST Forward* | GCCCAGCCCATTTCATTCAC |
| *RMST Reverse* | GCTCTGTCGTTCCACCTTGA |
| *PAR5 Forward* | GGTAGGAGGAGGGTTGGCTT |
| *PAR5 Reverse* | TTAGCAAGGCTGGACCTCAC |
| *IPW Forward* | TTTCTAGGTGAGCTGTTTGCCT |
| *IPW Reverse* | GAGTCCTGCCCAGCAAGATA |
| *PPIA Forward* | ATGCTGGACCCAACACAAAT |
| *PPIA Reverse* | TCTTTCACTTTGCCAAACACC |
| *EZH2 Forward* | CGCTTTTCTGTAGGCGATGT |
| *EZH2 Reverse*  *E-cadherin Forward*  *E-cadherin Reverse* | TGGGTGTTGCATGAAAAGAATA  CCCGGGACAACGTTTATTAC  GCTGGCTCAAGTCAAAGTCC |

Primers used for ChIP experiment

| **Primer name** | **Sequence 5'-3'** |
| --- | --- |
| *E-cadherin Forward* | GAACCCTCAGCCAATCAGC |
| *E-cadherin Reverse* | GAGTCTGAACTGACTTCCGC |

**Figure S2.** Originals blots for Western blot analyses in Figure 5. Western blot images of Figure 5C and Figure 5F relative to β-actin, H3K27me3, H3, E-cadherin and EZH2 in FRO and 8505c cell lines

**Figure S3.** Originals blots for Western blot analyses in Figure S1. Western blot images relative to β-actin and EZH2 in ATC and NT samples.
